# Evaluation of Cytologic Sample Preparations for Compatibility With Nucleic Acid Analysis

**DOI:** 10.1093/ajcp/aqab121

**Published:** 2021-09-20

**Authors:** Laure Sorber, Bart Claes, Karen Zwaenepoel, Bieke Van Dorst, Koen De Winne, Els Fransen, Reinier Wener, Therese Lapperre, Filip Lardon, Patrick Pauwels

**Affiliations:** 1 Center for Oncological Research (CORE), Integrated Personalized and Precision Oncology Network (IPPON), Wilrijk, Belgium; 2 Biocartis NV, Mechelen, Belgium; 3 miDIAGNOSTICS, Heverlee,Belgium; 4 Department of Pathology, University Hospital Antwerp (UZA), Edegem, Belgium; 5 Department of Pulmonology, University Hospital Antwerp (UZA), Edegem, Belgium; 6 Laboratory of Experimental Medicine and Paediatrics, University of Antwerp, Wilrijk, Belgium

## Abstract

**Objectives:**

In this study, the influence of several key elements of the cytologic sample workflow on DNA and RNA content was evaluated.

**Methods:**

The A549 cell line, patient-derived organoids, and pleural effusions were used to investigate the effect of (1) several collection media and delayed time to processing; (2) cytology specimens; (3) cytologic staining; and (4) formalin-fixed, paraffin-embedded (FFPE) cell block processing on nucleic acid quality and quantity as determined by fragment analyzer, Qubit analysis (Thermo Fisher Scientific), and quantitative polymerase chain reaction–based analysis on the Idylla platform (Biocartis).

**Results:**

Alcohol-based collection media (CytoRich Red [Thermo Fisher Scientific] and EtOH95%) displayed high DNA and RNA preservation capacity, while phosphate-buffered saline and, to a lesser extent, formalin were associated with high RNA quality. Cytospin and smear cytology specimens were subject to DNA and RNA loss. Cytologic staining had no further impact on sample quality, hence destaining is not necessary. Both H&E-stained and unstained FFPE sections are compatible with nucleic acid analysis, despite a strong decrease in DNA and RNA quality.

**Conclusions:**

Although several key elements of the cytologic sample workflow have an influence on DNA and RNA quality and quantity, the selection of these elements is also dependent on the downstream (ancillary) testing methods.

Key Points

- In lung cancer, cytologic samples are often the only source of material for molecular diagnostic testing. There are few studies on the influence of cytologic sample processing on DNA and RNA quality and quantity.
- Downstream (molecular) analyses largely determine the type of cytologic sample processing.
- Cytologic (May-Grünwald Giemsa, Papanicolaou) or H&E-stained samples were found to be compatible with fluorophore-based analysis. Hence, there is no need to destain samples.

## Introduction

Lung cancer is the most common malignancy and the leading cause of cancer-related death.^1^ The discovery of actionable oncogenic driver alterations combined with approved targeted therapies have significantly improved outcomes for patients with advanced-stage non–small cell lung cancer (NSCLC).^2^ It is currently standard practice in metastatic NSCLC to test for oncogenic drivers and biomarkers for immunotherapy—namely, *EGFR* alteration; *ALK*, *ROS1*, and *RET* rearrangements; *MET* amplification and *MET* exon 14 skipping; *BRAF* V600-sensitizing alteration; and programmed cell death 1 ligand 1 (PD-L1) expression.^3^ Recently, screening for *NTRK* fusions has also been included in the European Society for Medical Oncology recommendations.^4^ In many cases, only cytologic material is available for diagnostic, prognostic, and predictive assessment.^5^ There is wide variety in cytologic sample preparations and preanalytical factors, and their influence on the nucleic acid content is unclear. The College of American Pathologists (CAP) has developed a clinical practice guideline and recommendations for clinicians and pathologists on how to collect and process cytologic samples for ancillary testing.^6^ They graded their recommendations by the strength of the evidence because in some cases they were hampered by the paucity of high-quality studies on the computability of cytologic sample processing and ancillary testing. Furthermore, they took only DNA-based molecular testing into account, while RNA analysis may be better suited to fusion detection.^4^

In this study, the impact of several key elements of the cytologic sample workflow on DNA and RNA quality and quantity was evaluated, including collection media combined with delayed time to processing; different cytology specimens (cytospin and smear) and stains; and formalin-fixed, paraffin-embedded (FFPE) cell block processing. A lung cancer cell line, patient-derived organoids (PDOs), and pleural effusions (PEs) were used to obtain clinically relevant results. Remarkable differences were observed in sample quality; it was also noted that the choice of the preanalytical variables is subject to the downstream ancillary testing method.

## Materials and Methods

### Cell Culture and Sample Preparation

In this study, using a lung cancer cell line, 2 PDOs and leftover material of exudative PEs, the influence of several variables of the cytologic sample workflow on the quality and quantity of the genetic material was evaluated. In case of the PDO cell lines and PEs, ethical committee approval (20/06/059) was obtained. The study was conducted according to the Declaration of Helsinki.

The human lung cancer cell line A549, purchased from the American Type Culture Collection, was cultured as monolayers in Dulbecco’s Modified Eagle Medium (DMEM; Life Technologies), supplemented with 10% fetal bovine serum (Life Technologies), 1% penicillin (100 U/mL)/streptomycin (100 µg/mL; Life Technologies), and 2 mL L-glutamine (L-Glut; Life Technologies). Cells were maintained in exponential growth phase at 5% CO_2_ in a humidified incubator at 37°C. Cell cultures were regularly evaluated for the absence of mycoplasma contamination using the MycoAlert Mycoplasma Detection Kit (Lonza). Upon reaching an 80% confluence rate or higher, cells were harvested for downstream experiments by treatment with 0.25% trypsin for 5 minutes. Cells were resuspended in supplemented DMEM and centrifuged for 5 minutes at 450*g* (Centrifuge 5810R, Eppendorf). The supernatant was removed, and cells were resuspended in 1% phosphate-buffered saline (PBS). Cell counting (with trypan blue) was performed in triplicate using the TC20 Cell Counter (Bio-Rad).

The PDO cell lines were cultured according to the protocol of Dijstra et al^7^ with some minor alterations. The PDOs were collected from an established PDO cell line. For passaging, they were digested to single cells using TrypLE Express (Gibco). Afterwards, they were resuspended in Advanced DMEM/F12 medium supplemented with 1% GlutaMAX (Gibco), 1% penicillin/streptomycin, and 1% HEPES (ADF+++; Gibco), which in turn was supplemented with 10% Noggin-conditioned medium, 10% R-sponding-1-conditioned medium, b27 without vitamin A (Gibco), 10 mmol/L nicotinamide (Sigma-Aldrich), 25 ng/mL FGF-7 (Peprotech), 100 ng/mL FGF-10 (Peprotech), 500 nmol/L A83-01 (Tocris), and 5 µM Y-27632 (used only after passaging; Tocris). Single cells were embedded in basement membrane matrix (Cultrex Type 2; R&D Systems) and seeded on a preheated plate. After polymerization, the embedded cells were overlaid with the supplemented ADF+++ to facilitate organoid formation. Upon reaching a sufficient number of organoid-filled domes (confluence of 80% after 12-14 passages), the PDOs were harvested for downstream experiment by digesting them to single cells using TrypLE. They were resuspended in the supplemented ADF+++ and centrifuged for 5 minutes at 450*g.* The supernatant was removed, and cells were washed in PBS. Cell counting was performed in triplicate using the Cell Scepter (Scepter Cell Counter; Millipore).

Leftover material from exudative PEs of patients with lung cancer was collected in 50 mL conical centrifuge tubes (Falcon) and centrifuged for 5 minutes at 450*g.* The supernatant was removed, and cell pellets were combined and washed with PBS. The TC20 Cell Counter was used for cell counting. Threshold settings were adapted to exclude RBCs.

Cell suspensions of 200,000 cells per sample were prepared (unless otherwise specified). Samples were centrifuged for 5 minutes at 450*g* (Centrifuge 5417R; Eppendorf). The supernatant was removed, and the cell pellet was either immediately snap-frozen or used for downstream experiments.

### Evaluation Cytology Workflow

The focus of this study was to evaluate key elements of the cytologic sample workflow necessary to obtain adequate material for diagnostic testing.

### Collection Media and Delayed Time to Processing

As the first step after sample collection, several preservatives combined with a 72-hour delay in processing were evaluated on their compatibility with molecular analyses. In total, 6 collection media were selected based on literature review,^8-10^ a questionnaire data set, and considerations based on the new European regulation 2017/746 on in vitro diagnostics medical devices—namely, PBS, formaldehyde 4% buffered solution (formalin; Chem-Lab), ThinPrep CytoLyt Solution (Hologic), CytoRich Red preservative (Becton Dickinson), and ethanol solutions of 40% and 95% ethanol (EtOH40% and EtOH95%, respectively). The 72-hour delay in processing was selected to allow for samples left over the weekend.

We added 1 mL of collection medium to the cell pellet. Samples were left at room temperature for 72 hours before centrifugation for 5 minutes at 450*g.* The supernatant was removed, and the cell pellet was resuspended in PBS. Samples were centrifuged again, and the supernatant was removed. The cell pellet was snap-frozen in liquid nitrogen and stored at –80°C until nucleic acid isolation and analysis. All samples were processed in duplicate. Additionally, reference samples, consisting of cell pellets immediately snap-frozen after cell collection, were included.

### Cytology Specimens

Next, the adequacy of cytologic specimens (smears, cytospin) for nucleic acid–based testing was evaluated. Smear and cytospin specimens were compared with the reference samples. In case of smears, cell pellets were resuspended in approximately 10 µL PBS and pipetted onto a coated glass slide (microscope slides; Thermo Scientific). The samples were air-dried for up to 30 minutes before snap-freezing and storage at –80°C. Cytospin specimens were prepared by resuspending the cell pellets in approximately 300 µL PBS. The cytocentrifuge chamber was assembled by the sequential alignment of the cytofunnel (Shandon Single Cytofunnel with white filter cards; Thermo Scientific), a coated glass slide (Shandon Cytoslide; Thermo Scientific), and slide rack. The cell suspension was transferred into the cytofunnel and centrifuged for 5 minutes at 650*g* (Shandon Cytospin 3 cytocentrifuge; Thermo Scientific). Afterwards, the samples were air-dried for up to 30 minutes and snap-frozen.

### Cytologic Staining

The influence of cytologic staining methods on nucleic acid quantity and quality was assessed next. Papanicolaou and May-Grünwald Giemsa (Giemsa) stains were performed on cytospin specimens. The cell monolayer of these specimens will allow for optimal staining. Samples were fixated in CytoLyt for 1 hour before cytospin processing, as described in the section “Cytology Specimens.” Reference samples and CytoLyt-fixated samples (see “Collection Media and Delayed Time to Processing”) were also included. Cytospin specimens were allowed to air-dry for up to 30 minutes and stored at room temperature (RT) until staining. Papanicolaou and Giemsa staining (Sigma-Aldrich) was performed according to the manufacturer’s instructions. Cytospin specimens were air-dried for up to 1 hour before snap-freezing.

### FFPE Cell Blocks

The nucleic acid content of cell block specimens was investigated. Cell pellets consisting of 15 million to 20 million (A549 cell block) or more than 300 million cells (PE cell block) were centrifuged for 5 minutes at 450*g,* and the supernatant was removed. The cells were resuspended in formalin and incubated for 1 hour at RT. Afterwards, the samples were centrifuged again, and the majority of the supernatant was removed. The remaining supernatants were used to transfer the cell pellet into a mold containing approximately 200 µL or 1.5 mL preheated (60°C) 4% agar in the case of A549 or PE cell pellets, respectively. The mold was placed on a cold plate to allow the agar to solidify. The agar was placed in a cassette and loaded into the Excelsior ES Tissue Processor (Thermo Scientific). Processed paraffin blocks were stored at RT before sectioning. FFPE sections (5 µm; 6 sections per sample) were made to evaluate the effect of deparaffinization before nucleic acid isolation and H&E staining. For every 4 sections, 2 sections were taken for standard H&E staining.^11^ One H&E-stained section was used to evaluate cell content, while the other one, together with the remaining 2 unstained sections, was stored at –80°C until further processing.

### DNA and RNA Isolation

DNA and RNA were simultaneously isolated from all samples using the QIAamp DNA FFPE Tissue Kit (Qiagen), with some alterations to the protocol. The duration of the incubation steps at 56°C and 90°C were reduced from 1 hour to 15 and 10 minutes, respectively, based on an in-house protocol. In the case of FFPE sections, the original isolation protocol was used. Nucleic acids were eluted in 50 µL RNase-free water and stored at –80°C. The DNA and RNA isolation efficiency of the adapted protocol was compared with the standard protocol and the QIAamp Blood Mini Kit (Qiagen). The latter served as reference for the purification of nucleic acids from fresh samples (results described in Supplementary Data; all supplemental material can be found at *American Journal of Clinical Pathology* online).

Snap-frozen samples were thawed at 4°C for up to 30 minutes. The material of smeared, (stained) cytospin and FFPE specimens was scraped using a surgical scalpel. The scraping was facilitated by wetting the scalpel with lysis buffer. Before scraping, the H&E-stained and 1 of the unstained FFPE sections were deparaffined by subsequently immersing the sections in xylene and EtOH100%.

### DNA and RNA Analysis

The DNA and RNA concentrations of all samples were measured in duplicate using the Qubit 2.0 Fluorometer with the dsDNA HS Assay Kit and the RNA HS Assay Kit (Thermo Fisher Scientific), respectively, per the manufacturer’s instructions. Per duplicate sample set, the DNA and RNA integrity of 1 duplicate was evaluated using the HS Genomic DNA 50kb Kit and the HS RNA Kit (15NT), respectively, on the Fragment Analyzer (Applied Biosystems). Each sample was analyzed in duplicate according to the manufacturer’s instructions. Quality metrics were represented by the genomic quality number (GQN) and RNA quality number (RQN). We opted to use 2 GQNs based on the percentage of fragments of at least 10,000 and 200 base pairs (bp), respectively. The DNA and RNA quality of the other duplicate sample was assessed using a prototype version of the Idylla GeneFusion Assay on the Idylla Instrument (Biocartis), which contains 9 RNA targets—namely *ALK*, *ERCC3*, *MET*, *NTRK1/2/3*, *RET*, *ROS1,* and *TMUB2.* By experiment, a fixed amount of total RNA, ranging from 18 ng to 500 ng, was used.

### Statistical Analysis

The Kruskal-Wallis test was performed to ascertain whether there were significant differences in DNA and RNA quality and quantity between the various key elements of the cytologic workflow themselves and vs reference samples. The Mann-Whitney *U* test was used to further evaluate the effect between individual elements. Statistical analysis was performed using IBM SPSS Statistics, version 27, software. *P* < .05 was considered statistically significant. Figures were made in GraphPad Prism 7.

## Results

### Collection Media and Delayed Time to Processing

**Figure 1:**
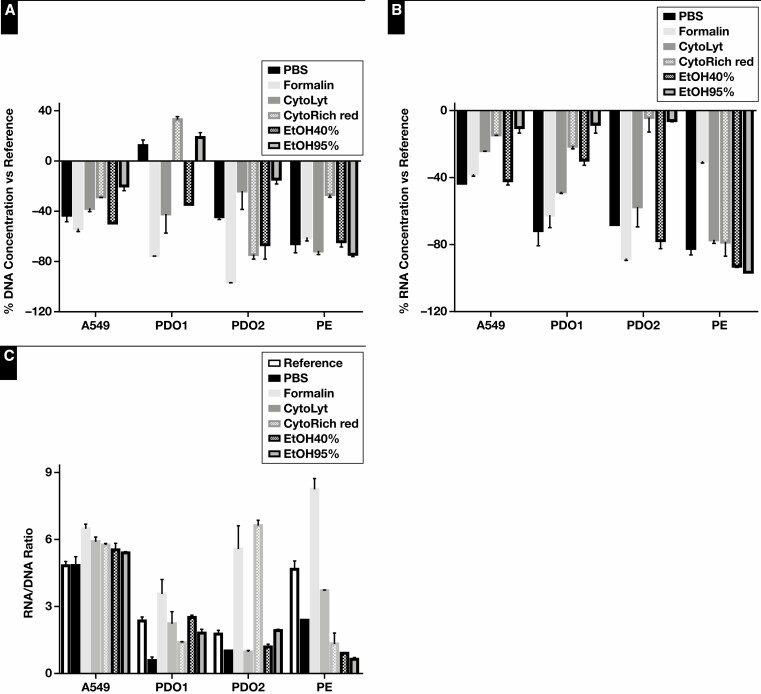
Collection media and delayed time to processing: DNA and RNA yield. The normalized DNA (**A**) and RNA (**B**) concentration and RNA-to-DNA ratio (**C**) of samples stored for 72 hours in various collection media. Immediately snap-frozen cell pellets served as reference. A549, A549 cell line; PBS, phosphate-buffered saline; EtOH, ethanol; PE, pleural effusion; PDO, patient-derived organoid.

**Figure 2:**
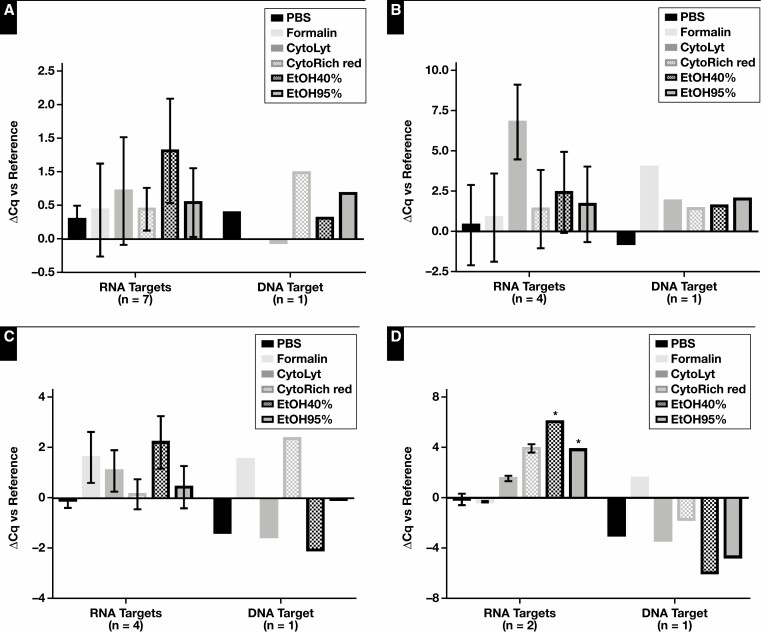
Collection media and delayed time to processing: DNA and RNA quality. The ΔCq values of samples stored for 72 hours in various collection media. DNA and RNA quality assessment was performed based on 1 DNA target and up to 7 RNA targets per sample type: A549 cell line (7 RNA targets) (**A**), patient-derived organoid (PDO) 1 (4 RNA targets) (**B**), PDO2 (4 RNA targets) (**C**), and pleural effusion (2 RNA targets) (**D**). Immediately snap-frozen cell pellets served as reference. PBS, phosphate-buffered saline; EtOH, ethanol. *Data from 1 RNA target.

In a first phase, the influence of several collection media (PBS, formalin, CytoLyt, CytoRich Red, EtOH40%, and EtOH95%) combined with delayed time to processing on DNA and RNA quality and quantity were evaluated. Significant differences were found in DNA concentration, RNA concentration, and the RNA-to-DNA ratio, which served as an indication of sample quality, between the reference and collection media samples in all cell types: A549 cell line, both PDOs, and PE. To further evaluate the influence of the various collection media, DNA and RNA yield was normalized to the reference  Figure 1  and data from all sample types were pooled. The DNA concentration varied among the different collection media (*P* = .021). It was found to be significantly lower in samples incubated in formalin vs PBS, CytoLyt, CytoRich Red, and EtOH95% (*P* = .009, *P* = .031, *P* = .021, and *P* = .021, respectively). This difference was less pronounced compared with EtOH40% (*P* = .059). The DNA concentration of samples incubated in EtOH40% also tended to be slightly lower compared with incubation in CytoRich Red and EtOH95% (*P* = .074 and *P* = .074, respectively). The RNA yield tended to vary less among the various collection media (*P* = .06). RNA concentrations were higher in samples collected in CytoRich Red than in PBS, formalin, and EtOH40% (*P* = .046, *P* = .026, and *P* = .021, respectively). The difference compared with CytoLyt was minor (*P* = .059). The RNA concentration of EtOH95% was similar to that of CytoRich Red. DNA quality assessed by Fragment Analyzer was also pooled for statistical analysis (Supplementary Table 2). The GQN (10,000) varied significantly between the reference and collection media samples (*P* = .036). No further statistical analysis was performed among the different sample types (A549, PDO1, PDO2, and PE) because of the limited data points. The DNA quality (GQN_10,000) of samples stored in PBS or CytoRich Red tended to be higher than in the other collection media across all sample types, except for PDO2. Although no significant differences in RNA quality were observed (*P* = .071), samples collected in formalin presented higher RQN scores (≥9.75 ± 0.25) across all sample types. A DNA fragment length of at least 200 bp was also retained in all samples and sample types. Sample quality in terms of quantitative polymerase chain reaction (qPCR) amplifiability was also assessed using the Idylla GeneFusion Assay  Figure 2 . In total, 1 DNA target and up to 7 RNA targets (A549 n = 7, PDOs n = 4, and PE n = 2) were used for DNA and RNA assessment, respectively. The ΔCq values of the collection media samples vs reference varied slightly in the A549 and PDO1 samples (*P* = .054 and *P* = .072, respectively). In the case of the PDO2 samples, significant variation was observed (*P* = .026). The difference was more significant when combining the data of all sample types (*P* = .007, based on 2 household RNA targets). The RNA quality of the samples collected in PBS was found to be significantly higher compared with samples collected in CytoLyt, CytoRich Red, EtOH40%, and EtOH95% (*P* = .006, *P* = .021, *P* = .008, and *P* = .005, respectively). No difference could be observed between samples collected in PBS or formalin, and the RNA quality of the latter was also significantly higher compared with EtOH40% (*P* = .015). The DNA quality was similar among the collection media samples.

### Cytology Specimens

**Figure 3:**
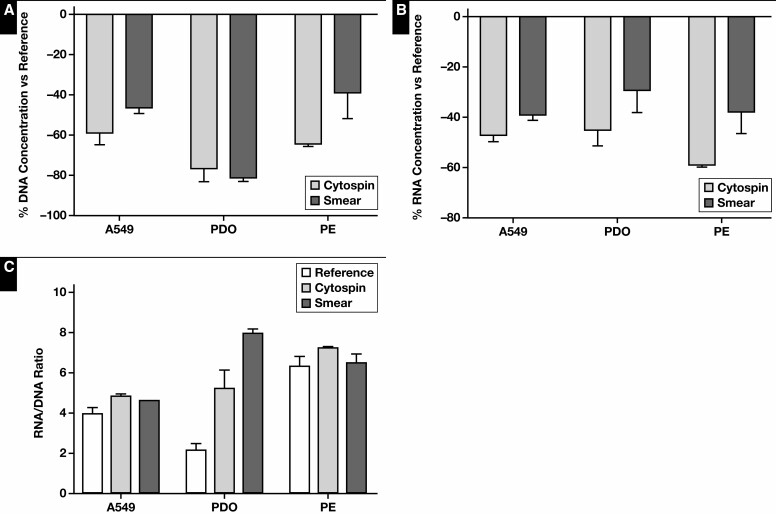
Cytology specimens: DNA and RNA yield. The normalized DNA (**A**) and RNA (**B**) concentration and RNA-to-DNA ratio (**C**) of cytospin and smear specimens. Immediately snap-frozen cell pellets served as reference. A549, A549 cell line; PDO, patient-derived organoid; PE, pleural effusion.

**Figure 4:**
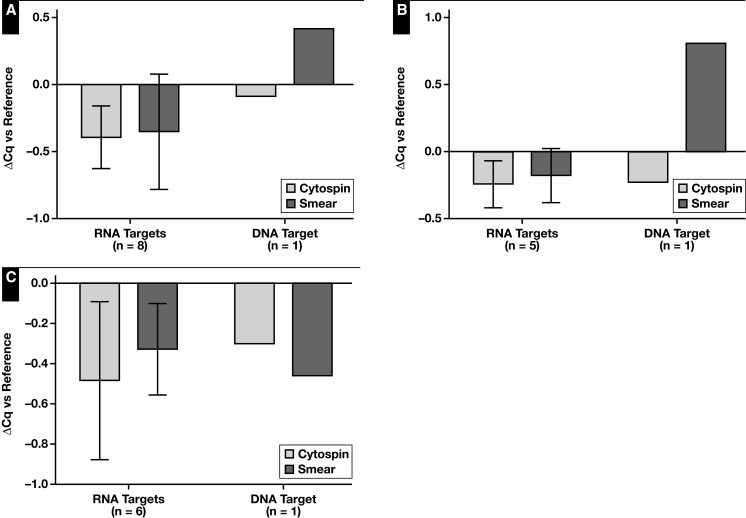
Cytology specimens—DNA and RNA quality: The ΔCq values of cytospin and smear specimens. DNA and RNA quality assessment was performed based on 1 DNA target and up to 8 RNA targets per sample type: A549 cell line (8 RNA targets) (**A**), patient-derived organoid (5 RNA targets) (**B**), and pleural effusion (6 RNA targets) (**C**). Immediately snap-frozen cell pellets served as reference.

Next, the effect of processing cytologic samples via cytospin or smear on the nucleic acid content was evaluated. In this experiment, the data of the Fragment Analyzer and Qubit analyses were pooled for statistical analysis  Figure 3 . A decrease in DNA and RNA concentration of the cytology specimens was observed compared with the reference (*P* = .06 and *P* = .05, respectively). Essentially, no statistically relevant difference in RNA-to-DNA ratio was observed (*P* = .073). The influence of the cytospin and smear processing itself was further investigated by normalizing the yield to the reference. Although the RNA yield of the cytospin specimens was lower compared with smear specimens (*P* = .016), no differences in DNA yield could be observed. A significant difference in DNA quality (based on GQN_10,000) was noted between the reference and cytology specimens (*P* = .027) (Supplementary Table 3). Because of the small sample size, no further statistical analyses were performed between these specimens. DNA (based on GQN_200) and RNA quality were similar. Quality assessment using the Idylla GeneFusion Assay revealed comparable findings  Figure 4 . In total, 1 DNA target and up to 8 RNA targets (A549 n = 8, PDO n = 5, and PE n = 6) were used. No differences in (Δ)Cq values for DNA and RNA were observed, and DNA quality was comparable among the cytology specimens.

### Cytologic Stains

**Figure 5:**
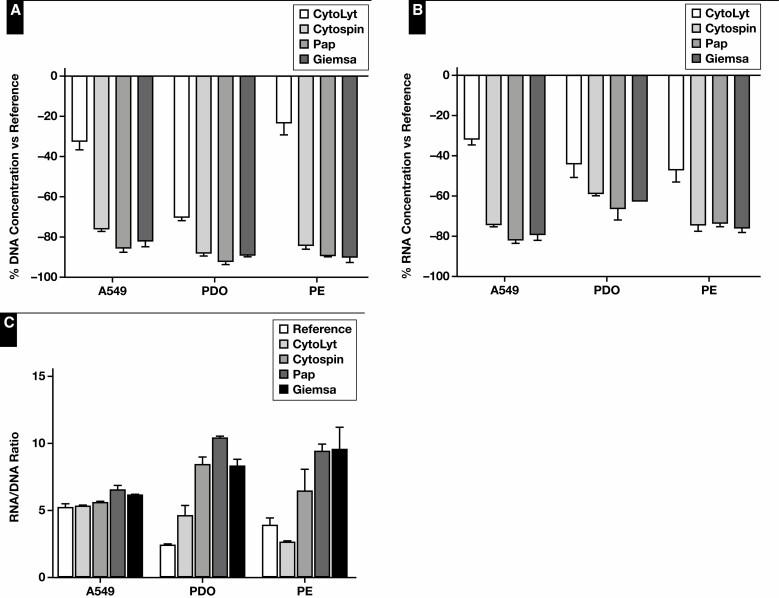
Cytologic staining: DNA and RNA yield. The normalized DNA (**A**) and RNA (**B**) concentration and RNA-to-DNA ratio (**C**) of several steps of the cytologic staining workflow. Immediately snap-frozen cell pellets served as reference. A549, A549 cell line; Giemsa, May-Grünwald Giemsa staining; Pap, Papanicolaou staining; PDO, patient-derived organoid; PE, pleural effusion.

**Figure 6:**
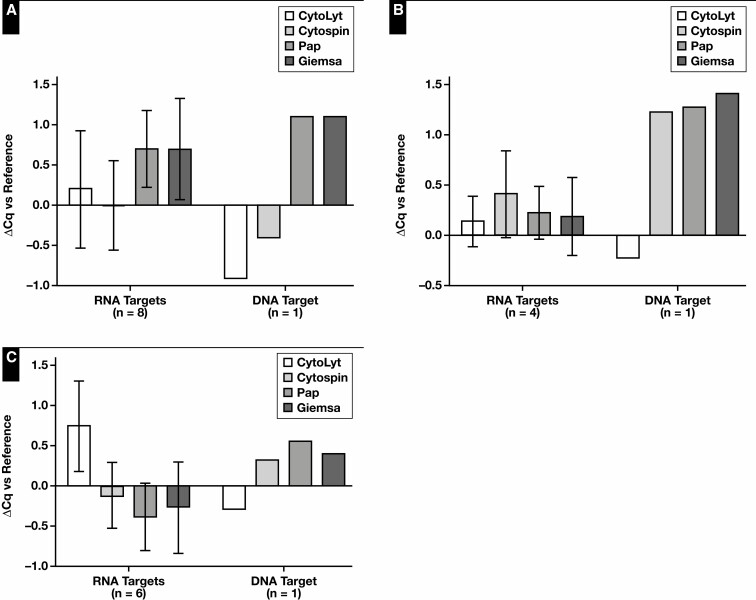
Cytologic staining: DNA and RNA quality. The ΔCq values of several steps of the cytologic staining workflow. DNA and RNA quality assessment was performed based on 1 DNA target and up to 8 RNA targets per sample type: A549 cell line (8 RNA targets) (**A**), patient-derived organoid (4 RNA targets) (**B**), and pleural effusion (6 RNA targets) (**C**). Immediately snap-frozen cell pellets served as reference. Giemsa, May-Grünwald Giemsa staining; Pap, Papanicolaou staining.

The steps of cytologic staining were evaluated for potential interference with DNA and RNA analyses. Fragment Analyzer and Qubit data were pooled for statistical analyses. The DNA and RNA concentrations as well as the RNA-to-DNA ratio were found to differ significantly among all samples (*P* < .001, *P* = .002, and *P <* .001, respectively). These differences were also observed among the cytologic staining process samples themselves, except for the RNA concentration (DNA *P* = .002, RNA *P* = .054, and RNA-to-DNA ratio *P* = .003). To further evaluate the effect of the cytologic staining process, the DNA and RNA yield was normalized to the reference  Figure 5 . The effects of the cytologic staining process on the DNA and RNA yield were significant (*P* = .001 and *P* = .003, respectively) compared with the reference sample. The DNA and RNA concentration of the stained and unstained cytospin specimens was similar. Only in the case of the Papanicolaou-stained specimen was the DNA concentration lower than its unstained counterpart (*P* = .037). The DNA and RNA concentration of the CytoLyt-fixated samples was significantly higher than the stained and unstained cytospin specimens (*P* < .001 in all cases). A trend was observed in DNA quality (based on GQN_10,000) between the reference and cytologic staining samples (*P* = .062) (Supplementary Table 4). No significant differences in DNA (based on GQN_200) and RNA quality among these samples could be observed. The DNA and RNA quality was also assessed using the Idylla GeneFusion Assay  Figure 6  using 1 DNA target and up to 8 RNA targets (A549 n = 8, PDO n = 4, and PE n = 6). Although the ΔCq values varied among the cytologic staining process samples of the A549 and PE sample types (*P* = .02 and *P* = .021, respectively), this was no longer observed when the data of the sample types were combined (based on 3 common RNA targets). DNA quality was also similar between the cytologic staining process samples.

### FFPE Cell Blocks

**Figure 7:**
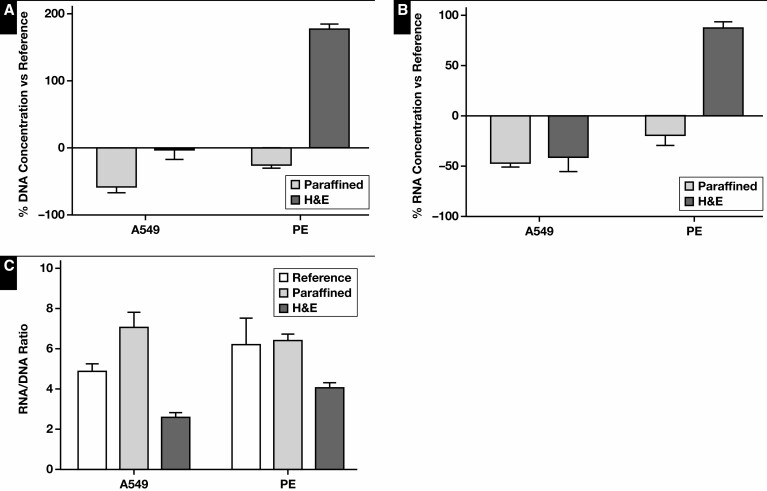
Formalin-fixed, paraffin-embedded (FFPE) cell blocks: DNA and RNA yield. The normalized DNA (**A**) and RNA (**B**) concentration and RNA-to-DNA ratio (**C**) of paraffin- and H&E-stained fresh-frozen FFPE sections. Deparaffined FFPE sections served as reference. A549, A549 cell line; PE, pleural effusion.

**Figure 8:**
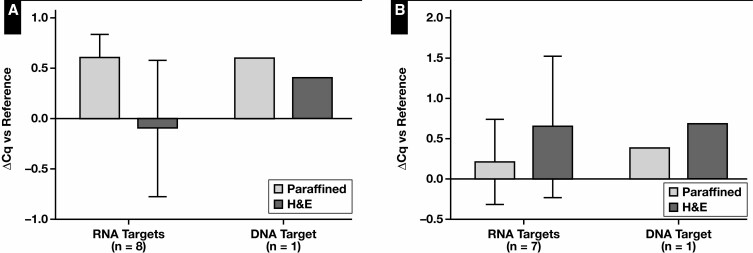
Formalin-fixed, paraffin-embedded (FFPE) cell blocks: DNA and RNA quality. The ΔCq values of paraffin- and H&E-stained fresh-frozen FFPE sections. DNA and RNA quality assessment was performed based on 1 DNA target and up to 8 RNA targets per sample type: A549 cell line (8 RNA targets) (**A**) and pleural effusion (7 RNA targets) (**B**). Deparaffined FFPE sections served as reference.

Finally, the DNA and RNA quality and quantity of FFPE cell block specimens were evaluated. Because A549 characterizes cell-poor specimens and PE FFPE cell blocks are cell-rich specimens, we opted not to combine the data; thus, no statistical analysis was performed. The yield of unstained paraffin and H&E-stained sections was compared with unstained, deparaffined sections, which served as reference  Figure 7 . The paraffin sections produced lower DNA and RNA concentrations than their deparaffined counterparts. Interestingly, this was not the case with H&E-stained sections. In the case of the PE FFPE cell block, the DNA and RNA concentrations were even higher than the reference deparaffined sections. This discrepancy between the A549 and PE FFPE cell blocks was also observed in DNA and RNA quality, as determined by Fragment Analyzer analysis (Supplementary Table 5). The H&E-stained sections appeared to have the lowest sample quality in the A549 cell blocks and the highest sample quality in PE FFPE cell blocks. In contrast to the previous experiments, lower GQN scores based on 200 bp were noted for all FFPE sections. Sample quality based on RQN and GQN (10,000 and 200) was noted to be significantly lower compared with the other experiments (*P* = .002, *P* = .003, and *P* < .0001, respectively). Quality assessment using the Idylla GeneFusion Assay, however, revealed that the amplification reactions were not impaired in any sample type or specimen  Figure 8 .

## Discussion

Cytologic samples have found their way in routine molecular testing for the selection of patients who may benefit from targeted therapy. Originally, guidelines from the CAP, the International Association for the Study of Lung Cancer (IASLC), and the Association for Molecular Pathology (AMP) recommended using only cell blocks for molecular testing. Several studies, however, as well as evidence from real-world clinical practice have revealed that any cytologic sample with adequate cellularity and preservation can be used for ancillary testing.^12,13^ In this study, the influence of several key elements of the cytologic sample workflow on the compatibility of these samples with ancillary testing was investigated. Besides a lung cancer cell line, PDOs and PEs were used to ensure clinically relevant findings.

First, the influence of several collection media (PBS, formalin, CytoLyt, CytoRich Red, EtOH40%, and EtOH95%) and delayed time to processing was investigated. CytoRich Red was found to preserve the RNA content significantly better than the other collection media. Only samples collected in EtOH95% had a similar RNA yield. Samples collected in formalin had the lowest DNA yield, followed by EtOH40%. In contrast, CytoRich Red and EtOH95% had the highest DNA preservation capacity. The DNA quality of samples collected in CytoRich Red and EtOH95% seemed somewhat higher than the other collection media. This phenomenon was not observed with RNA quality, which was significantly higher in samples stored in PBS and, to a lesser degree, in formalin. The high RNA quality of the latter was surprising because studies have shown higher-quality nucleic acids in noncross-linking alcoholic reagents.^8^ Formalin is one of the most commonly used fixatives for both tissue and cytologic samples as part of the paraffin embedding process and is known to induce methylene bridging of bases and the formation of cross-links.^10^ The low RNA quality of FFPE sections is partly the result of the relatively harsh conditions of RNA isolation—namely, the proteinase K digestion followed by the heating steps to remove these chemical modifications.^14^ The cell pellets in this study had not undergone paraffin embedding; thus, the duration of the proteinase K digestion and heating steps has been decreased, resulting in high RNA quality. The lower DNA yields are most likely the result of incomplete removal of the chemical modifications by the adapted isolated protocol rather than the low DNA preservation capacity of formalin. Interestingly, CytoRich Red contains a small percentage (<1%) of formaldehyde, resulting in cross-linking. Dejmek et al^15^ reported poorer DNA preservation by CytoRich Red compared with CytoLyt, despite both collection media being alcohol based. This behavior was not observed in this study, with CytoRich Red even (slightly) outperforming CytoLyt in DNA and RNA preservation. This discrepancy may be explained by Dejmek et al^15^ using only cell lines. Furthermore, the DNA yield was determined after cell pellets collected in CytoRich Red were deposited on SurePath PreCoat slides (TriPath Imaging) and stained with H&E or Papanicolaou staining, possibly masking the influence of CytoRich Red itself. EtOH95% was also found to have good RNA and DNA preservation capacity, as noncross-linking alcoholic reagents are known to cause little chemical changes.^8,16,17^ The lower preservation capacity observed in EtOH40% is possibly the result of the minor ethanol percentage combined with the fact that pure water is not an ideal medium for cells. The choice of collection medium will most likely depend on the downstream processing methods and ancillary testing. Despite rather limited evidence, the CAP does recommend collecting cytology samples in formalin,^6^ the most commonly used fixative for the generation of FFPE (cell) blocks.^8,16,18^ Although FFPE cell blocks can be generated from specimens fixated in alcohol-based collection media, a postfixation step in formalin is typically performed. This step might reduce the quality of the often-limited material even more.^10^ In case of PCR-based analysis, however, both this study and others^8,16,17^ demonstrate that alcohol-based collection media (eg, EtOH95% and CytoRich Red) may be a good alternative. Because all samples were washed with PBS before snap-freezing and the subsequent nucleic acid isolation, the impact of this agent on isolation efficiency^16^ was not considered while evaluating the effect of the collection media on DNA and RNA.

In the next phase, the sample quality of cytospin and smear specimens was evaluated. No significant difference in DNA and RNA quantity or quality could be observed between the 2 specimens, but it seems that both had a marked decrease in DNA and RNA concentration compared with the reference sample. It is likely that cells or cell content was lost during collection, despite the use of coated slides and collection via scalpel-blade scraping, which has been shown to yield higher DNA concentrations than cell lifting.^19^ It is also important to note that no coverslip was used on these specimens. If a coverslip is present, it can be removed by soaking in xylene^20^ or the “freezer” method.^21^ This step reportedly has no influence on DNA retrieval, but the effects on RNA are still unknown.

Next, the compatibility of Giemsa- and Papanicolaou-stained slides with molecular testing was evaluated. Samples were collected throughout the cytologic staining process. As seen in the previous section, there was a marked decrease in DNA and RNA concentration of the (un)stained cytospin specimens compared with the reference and the CytoLyt-fixed samples. The DNA yield of Papanicolaou-stained specimens was lower than the Giemsa- and unstained counterparts. No significant differences in RNA concentration or DNA and RNA quality were detected. These data correspond to previously reported findings that Papanicolaou-stained specimens are compatible with DNA-based molecular testing.^15,22-24^ In 2 of those studies, it was found that Papanicolaou-stained specimens had a (slightly) lower DNA yield than their Diff-Quik–stained counterparts,^22,24^ and another study demonstrated higher DNA fragmentation in archival (>10 years of age) Papanicolaou-stained specimens.^23^ Both Diff-Quik and Giemsa are Romanowsky stains, the former being a simplified modification, which may explain the similar findings reported in this study. Papanicolaou and Giemsa contain strong chromophores,^25^ such as hematoxylin and eosin. No interference with the fluorophore-based Idylla qPCR system was observed because these chromophores are likely not retained in the final eluate. Hence, there is no need to destain these specimens before fluorophore-based analyses.

Finally, we evaluated the sample quality of FFPE specimens. One FFPE cell block was constituted as a cell-poor (A549) and the other as a cell-rich specimen (PE). The effect of deparaffinization and H&E staining was evaluated by cell block. The DNA and RNA concentrations were higher in H&E-stained sections A549 and PE cell blocks than in the unstained counterparts. This observation was more remarkable in samples from the PE cell block. To make cell blocks, the cell pellets must be held in place. In this study, agar was used. Other agents, such as plasma thrombin, gelatin, and HistoGel, are available, as well.^26^ It is likely that the agar was dissolved during the H&E staining process. It was noted that during isolation, the columns of the unstained, deparaffined sections of the PE cell block became slightly clogged, while this was not the case with H&E-stained sections. A possible explanation is that agar is not dissolved completely by the deparaffinization process, allowing it to clot after the 90°C incubation step and capturing or preventing nucleic acids from going through the column membrane. In case of paraffin sections, this was even more substantial because of the presence of both the paraffin and agar, resulting in low DNA and RNA concentrations. Because the PE cell block was extremely cell rich, a larger mold and more agar were necessary to set the cell pellet. Hence, the effect of the H&E staining and the agar itself was observed more clearly in the PE cell block specimens than in the A549 specimens. In contrast, DNA and RNA quality and amplification were similar among all FFPE specimens. The significantly lower RQN and GQN scores of the FFPE samples compared with the other specimens in this study clearly highlighted the DNA and RNA degradation associated with FFPE material.^9,16,18^ Despite this finding, cell blocks remain the cytologic substrate of choice. Ancillary molecular methods, such as fluorescence in situ hybridization and immunohistochemistry (IHC), have been validated on FFPE tissue blocks.^6,27^ Cell block is also suggested as a valuable sample type because it allows the performance of multiple IHC markers for diagnostics (eg, histologic [sub]typing) and predictive (eg, PD-L1) assessment.^28^ Several studies indicate that alcohol-based fixated cytology samples (eg, smears and liquid-based preparations) are better suited for PCR-based assessment than FFPE cell blocks,^16,17^ but these findings on cytologic stains suggest that the presence of H&E did not hamper analysis with the fluorophore-based Idylla system.

## Conclusions

The alcohol-based collection media CytoRich Red and EtOH95% seem to have the highest DNA and RNA preservation capacity, with limited quality decrease. Despite this, these cell pellets as such are rarely used in clinical practice because they do not allow histologic assessment. They are most often processed as cytology specimens or FFPE cell blocks. Processing cytologic samples as cytospin or smear specimen was found to result in a significant decrease of DNA and RNA concentration, most likely because of the procedure of cell collection from both specimens. Interestingly, the cytologic stains Papanicolaou and Giemsa did not further influence DNA and RNA concentration or quality. They were even found to be compatible with fluorophore-based analysis despite containing strong chromophores. In the case of FFPE cell blocks, the use of formalin as collection medium is recommended to avoid an additional fixation step in formalin before paraffin embedding. Despite a strong decrease in DNA and RNA quality, H&E-stained and unstained (with or without deparaffinization) FFPE sections were found to be compatible with nucleic acid analysis. Ultimately, the selection of preanalytical variables is largely dependent on downstream ancillary testing.

## Supplementary Material

aqab121_suppl_Supplementary_Material

## Notes

Funding: This work was supported by VLAIO (Vlaanderen—Agentschap Innoveren & Ondernemen) [HBC.2020.2173].
