# Supplementary material for: Evaluation of Cytologic Sample Preparations for Compatibility With Nucleic Acid Analysis
Source: Am J Clin Pathol. 2021 Sep 20;157(2):293–304. doi: 10.1093/ajcp/aqab121 (PMC8824667; doi:10.1093/ajcp/aqab121)
Supplement: aqab121_suppl_Supplementary_Material [file aqab121_suppl_supplementary_material.docx]

**SUPPLEMENTARY DATA**

To minimize variability between reference (fresh material) and processed samples (possibly fixated), the protocol of the QIAamp® DNA FFPE Tissue kit (Qiagen; FTK_adapt) was adapted to efficiently isolate both sample types. The DNA and RNA isolation efficiency was compared to the standard protocol of the kit (FTK) as well as the QIAamp® Blood Mini kit (Qiagen), which served as reference. The results of the Fragment Analyzer and qubit analyses of the A549 cell line, patient-derived organoids (PDO), and pleural effusion (PE) were combined for statistical analysis. As the total DNA and RNA concentrations differed per cell type (*p* = 0.002 and *p* = 0.001, respectively), the yield of FTK and FTK_adapt were normalized to the reference (Supplementary figure 1). The DNA and RNA isolation efficiency of FTK_adapt was found to be significantly improved compared to FTK (*p* = 0.01 and *p* = 0.01, respectively). The quality of the isolated RNA and DNA was also evaluated (Supplementary Table 1). A significant difference in RQN (*p* = 0.038) was found between all of the isolation kits. Due to the small sample number, no further statistical analyses were performed between the different kits. A trend in GQN, based on a 10 000-base pair (bp) threshold (*p* = 0.066) was also observed. There were no differences in GQN based on 200-bp or RNA versus DNA ratio. Based on these findings, FTK_adapt was used to isolate DNA and RNA from all samples in this study, with the exception of formalin-fixed paraffin embedded (FFPE) samples.


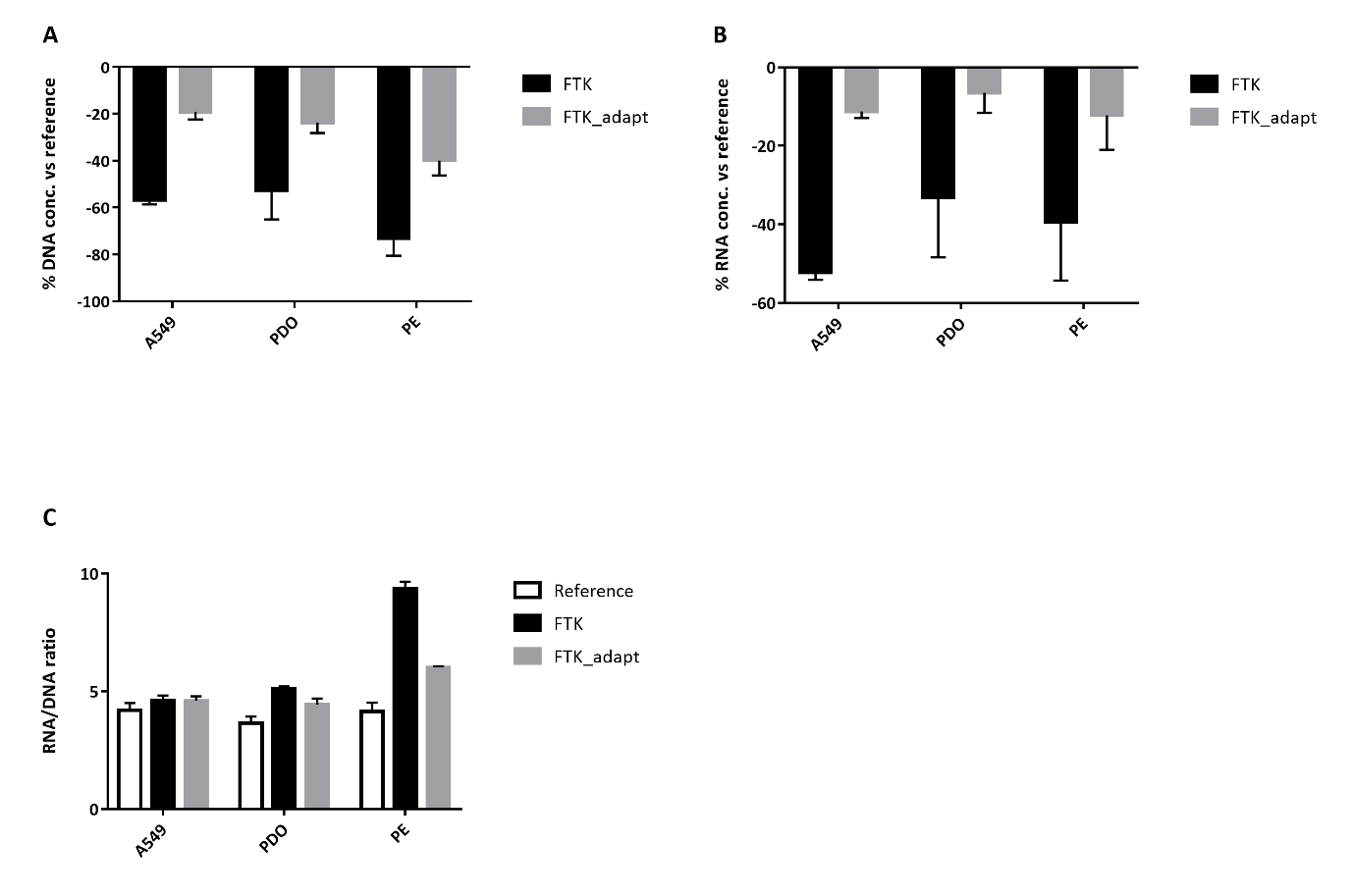


*Supplementary figure 1:* Adaptation isolation protocol – DNA and RNA yield

The normalized DNA (**A**) and RNA (**B**) concentration and RNA vs DNA ratio (**C**) of samples processed with the standard and adapted FTK protocols. The reference consisted of samples processed with the QIAamp® Blood Mini kit.

*A549: A549 cell line, FTK: QIAamp® DNA FFPE Tissue Kit; FTK_adapt: adapted protocol of FTK; PE: pleural effusion, PDO: patient-derived organoids.*

| Supplementary Table 1: Adaptation isolation kit – DNA and RNA quality | | | |
| --- | --- | --- | --- |
| RQN | | | |
|  | **A549** | **PDO** | **PE** |
| Reference | 10 ± 0 | 10 ± 0 | 8.9 ± 0.4 |
| FTK | 3 ± 0.8 | 4 ± 0 | 1.15 ± 0.06 |
| FTK_adapt | 3.3 ± 1.1 | 6.45 ± 0.55 | 5.93 ± 0.9 |
| GQN (10 000) | | | |
|  | **A549** | **PDO** | **PE** |
| Reference | 2.6 ± 0 | 3.8 ± 0.2 | 2.5 ± 0.06 |
| FTK | 0.5 ± 0 | 0.93 ± 0.12 | 0.9 ± 0 |
| FTK_adapt | 1.5 ± 0.1 | 2.9 ± 0.12 | 2.73 ± 0.09 |
| GQN (200) | | | |
|  | **A549** | **PDO** | **PE** |
| Reference | 9.8 ± 0 | 9.97 ± 0.03 | 10 ± 0 |
| FTK | 8.15 ± 0.05 | 9.47 ± 0.48 | 10 ± 0 |
| FTK_adapt | 9.7 ± 0 | 9.98 ± 0.03 | 9.9 ± 0 |
| *A549: A549 cell line; GQN: genomic quality number; FTK: QIAamp® DNA FFPE Tissue Kit; FTK_adapt: adapted protocol of FTK; RQN; PE: pleural effusion; PDO: patient-derived organoids; RNA quality number* | | | |

| Supplementary Table 2: Collection media and delayed time-to-processing – DNA and RNA quality | | | | |
| --- | --- | --- | --- | --- |
| RQN | | | | |
|  | **A549** | **PDO1** | **PDO2** | **PE** |
| Reference | 9 ± 0.74 | 5 ± 0.95 | 9.2 ± 0.7 | 3.64 ± 0.54 |
| PBS | 6.3 ± 3.7 | 8.95 ± 0.05 | NA | 5.85 ± 0.83 |
| Formalin | 10 ± 0 | 9.75 ± 0.25 | 9.45 ± 0.25 | 9.4 ± 0.55 |
| CytoLyt® | 2.5 ± 1.5 | 4.9 ± 2.59 | 10 ± NA | 6.1 ± 0.4 |
| CytoRich™ Red | 4.8 ± 0.1 | 3.75 ± 1.34 | 8.1 ± 0 | 1.15 ± 0.15 |
| EtOH40% | 1.75 ± 0.75 | 1.2 ± 0.14 | NA | 9.35 ± 0.32 |
| EtOH95% | 1.75 ± 0.15 | 2.85 ± 0.64 | 5.95 ± 2.85 | 4.95 ± 3.95 |
| GQN (10 000) | | | | |
|  | **A549** | **PDO1** | **PDO2** | **PE** |
| Reference | 2.95 ± 0.1 | 2.8 ± 0.1 | 5.4 ± 0.1 | 2.75 ± 0.15 |
| PBS | 2.45 ± 0.05 | 7.8 ± 0.7 | 5.65 ± 0.25 | 4.5 ± 1 |
| Formalin | 0.2 ± 0 | 1.15 ± 0.25 | 1.6 ± 0.1 | 0.7 ± 0.3 |
| CytoLyt® | 0.45 ± 0.05 | 0.5 ± 0 | 3.15 ± 0.05 | 1.15 ± 0.25 |
| CytoRich™ Red | 2.25 ± 0.15 | 3.75 ± 0.45 | 0.9 ± 0 | 6.45 ± 0.15 |
| EtOH40% | 1.75 ± 0.05 | 1.55 ± 0.05 | 5.15 ± 0.25 | 1.1 ± 0.3 |
| EtOH95% | 1.8 ± 0 | 2.35 ± 0.05 | 5.65 ± 0.35 | 7.3 ± 0.2 |
| GQN (200) | | | | |
|  | **A549** | **PDO1** | **PDO2** | **PE** |
| Reference | 9.98 ± 0.03 | 10 ± 0 | 10 ± 0 | 10 ± 0 |
| PBS | 9.95 ± 0.05 | 10 ± 0 | 10 ± 0 | 10 ± 0 |
| Formalin | 9.95 ± 0.05 | 10 ± 0 | 9.9 ± 0 | 9.9 ± 0 |
| CytoLyt® | 9.85 ± 0.05 | 9.95 ± 0.05 | 10 ± 0 | 10 ± 0 |
| CytoRich™ Red | 10 ± 0 | 10 ± 0 | 10 ± 0 | 10 ± 0 |
| EtOH40% | 9.85 ± 0.05 | 9.95 ± 0 | 10 ± 0 | 10 ± 0 |
| EtOH95% | 9.8 ± 0 | 9.9 ± 0 | 10 ± 0 | 10 ± 0 |
| *A549: A549 cell line; GQN: genomic quality number; NA: data not available; RQN; PE: pleural effusion; PDO: patient-derived organoids; RNA quality number* | | | | |

| Supplementary Table 3: Cytology specimens – DNA and RNA quality | | | |
| --- | --- | --- | --- |
| RQN | | | |
|  | **A549** | **PDO** | **PE** |
| Reference | 8.25 ± 0.05 | 8.5 ± 0.3 | 3.9 ± 0.4 |
| Cytospin | 6.6 ± 0.1 | 6.65 ± 0.15 | 5.65 ± 0.15 |
| Smear | 6.4 ± 0.1 | 6.25 ± 0.05 | 5.6 ± 0.8 |
| GQN (10 000) | | | |
|  | **A549** | **PDO** | **PE** |
| Reference | 3.4 ± 0.4 | 6.6 ± 0.2 | 3.55 ± 0.05 |
| Cytospin | 2.2 ± 0 | 3.05 ± 0.15 | 1.95 ± 0.15 |
| Smear | 1.75 ± 0.05 | 1.9 ± 0.1 | 1.65 ± 0.25 |
| GQN (200) | | | |
|  | **A549** | **PDO** | **PE** |
| Reference | 10 ± 0 | 9.95 ± 0.05 | 10 ± 0 |
| Cytospin | 10 ± 0 | 10 ± 0 | 9.95 ± 0.05 |
| Smear | 10 ± 0 | 9.9 ± 0.1 | 10 ± 0 |
| *A549: A549 cell line; GQN: genomic quality number; RQN; PE: pleural effusion; PDO: patient-derived organoids; RNA quality number* | | | |

| Supplementary Table 4: Cytological staining – DNA and RNA quality | | | |
| --- | --- | --- | --- |
| RQN | | | |
|  | **A549** | **PDO** | **PE** |
| Reference | 8.95 ± 0.05 | 9.53 ± 0.48 | 5.45 ± 0.05 |
| CytoLyt® | 8.65 ± 0.05 | 7.55 ± 0.05 | 5.8 ± 0.2 |
| Cytospin | 6.3 ± 0.8 | 4.4 ± 0.6 | 1.8 ± 0.6 |
| PAP | 3.75 ± 0.35 | 4.43 ± 0.98 | 2.85 ± 1.05 |
| Giemsa | 6.15 ± 0.95 | 6.7 ± 1.39 | 1.15 ± 0.05 |
| GQN (10 000) | | | |
|  | **A549** | **PDO** | **PE** |
| Reference | 3.55 ± 0.05 | 6.3 ± 0.33 | 2.9 ± 0.3 |
| CytoLyt® | 1.8 ± 0 | 5.2 ± 0.3 | 5.25 ± 0.15 |
| Cytospin | 0.85 ± 0.05 | 1 ± 0.1 | 2.45 ± 0.15 |
| PAP | 0.45 ± 0.05 | 0.85 ± 0.05 | 1.85 ± 0.05 |
| Giemsa | 0.15 ± 0.05 | 1.3 ± 0.15 | 1.2 ± 0.2 |
| GQN (200) | | | |
|  | **A549** | **PDO** | **PE** |
| Reference | 10 ± 0 | 10 ± 0 | 9.95 ± 0.05 |
| CytoLyt® | 10 ± 0 | 10 ± 0 | 10 ± 0 |
| Cytospin | 10 ± 0 | 9.8 ± 0 | 9.9 ± 0 |
| PAP | 9.95 ± 0.05 | 9.95 ± 0.05 | 9.9 ± 0 |
| Giemsa | 10 ± 0 | 10 ± 0 | 9.85 ± 0.05 |
| *A549: A549 cell line; Giemsa: May-Grünwald Giemsa staining; GQN: genomic quality number; RQN; PAP: Papanicolaou staining; PE: pleural effusion; PDO: patient-derived organoids; RNA quality number* | | | |

| Supplementary Table 5: FFPE cell blocks – DNA and RNA quality | | |
| --- | --- | --- |
| RQN | | |
|  | **A549** | **PE** |
| Reference | - 1. ± 0.18 | 2.4 ± 0 |
| Paraffin | 1.3 ± 0 | 2.1 ± 0.2 |
| H&E | 2.3 ± 0.3 | 1.95 ± 0.45 |
| GQN (10 000) | | |
|  | **A549** | **PE** |
| Reference | 0.28 ± 0.08 | 1 ± 0.3 |
| Paraffin | 0.35 ± 0.25 | 1.4 ± 0.5 |
| H&E | 0.6 ± 0 | 0.28 ± 0.09 |
| GQN (200) | | |
|  | **A549** | **PE** |
| Reference | 5.88 ± 0.03 | 9.55 ± 0.05 |
| Paraffin | 5.45 ± 0.25 | 9.2 ± 0.1 |
| H&E | 7.4 ± 0 | 8.85 ± 0.21 |
| *A549: A549 cell line; GQN: genomic quality number; H&E:* *hematoxylin and eosin staining; RQN; PE: pleural effusion; PDO: patient-derived organoids; RNA quality number* | | |
